# Rapid excision of oxidized adenine by human thymine DNA glycosylase

**DOI:** 10.1016/j.jbc.2022.102756

**Published:** 2022-11-30

**Authors:** Hardler W. Servius, Lakshmi S. Pidugu, Matthew E. Sherman, Alexander C. Drohat

**Affiliations:** Department of Biochemistry and Molecular Biology, University of Maryland School of Medicine, Baltimore, Maryland, USA

**Keywords:** base excision repair, DNA damage, DNA repair, oxidation, 7,8-dihydro-8-oxoadenine, nucleic acid chemistry, enzyme, εC, 3,*N*^4^-ethenocytosine, caC, 5-carboxylcytosine, fC, 5-formylcytosine, MUG, mismatch uracil glycosylase, ODN, oligodeoxynucleotide, oxoA, 7,8-dihydro-8-oxoadenine, oxoG, 7,8-dihydro-8-oxoguanine, Pol I, DNA polymerase I, Pol α, DNA polymerase alpha, Pol β, DNA polymerase beta, Pol η, DNA polymerase eta, TDG, thymine DNA glycosylase

## Abstract

Oxidation of DNA bases generates mutagenic and cytotoxic lesions that are implicated in cancer and other diseases. Oxidative base lesions, including 7,8-dihydro-8-oxoguanine, are typically removed through base excision repair. In addition, oxidized deoxynucleotides such as 8-oxo-dGTP are depleted by sanitizing enzymes to preclude DNA incorporation. While pathways that counter threats posed by 7,8-dihydro-8-oxoguanine are well characterized, mechanisms protecting against the major adenine oxidation product, 7,8-dihydro-8-oxoadenine (oxoA), are poorly understood. Human DNA polymerases incorporate dGTP or dCTP opposite oxoA, producing mispairs that can cause A→C or A→G mutations. oxoA also perturbs the activity of enzymes acting on DNA and causes interstrand crosslinks. To inform mechanisms for oxoA repair, we characterized oxoA excision by human thymine DNA glycosylase (TDG), an enzyme known to remove modified pyrimidines, including deaminated and oxidized forms of cytosine and 5-methylcystosine. Strikingly, TDG excises oxoA from G⋅oxoA, A⋅oxoA, or C⋅oxoA pairs much more rapidly than it acts on the established pyrimidine substrates, whereas it exhibits comparable activity for T⋅oxoA and pyrimidine substrates. The oxoA activity depends strongly on base pairing and is 370-fold higher for G⋅oxoA *versus* T⋅oxoA pairs. The intrinsically disordered regions of TDG contribute minimally to oxoA excision, whereas two conserved residues (N140 and N191) are catalytically essential. *Escherichia coli* mismatch-specific uracil DNA-glycosylase lacks significant oxoA activity, exhibiting excision rates 4 to 5 orders of magnitude below that of its ortholog, TDG. Our results reveal oxoA as an unexpectedly efficient purine substrate for TDG and underscore the large evolutionary divergence of TDG and mismatch-specific uracil DNA-glycosylase.

The nucleobases of DNA are highly susceptible to oxidation, generating a plethora of mutagenic and cytotoxic lesions that threaten genetic integrity and are implicated in cancer and other diseases as well as aging (1). Perhaps the most prominent example is 7,8-dihydro-8-oxoguanine (oxoG), the major product of guanine oxidation (2). The threat posed by oxoG and other oxidative lesions is countered largely by base excision repair (3, 4), a pathway initiated by DNA glycosylases that cleave the *N*-glycosyl bond to remove modified bases. Adenine is also subject to oxidation, and a major product is 7,8-dihydroxy-8-oxoadenine (oxoA) (Fig. 1*A*). The enol tautomer of this lesion, 8-hydroxyadenine, appears in the early literature, but the 6-amino-8-keto species is the predominant form in aqueous solution (5, 6). oxoA is generated by ionizing irradiation and other oxidizing agents, and the adenine lesion is readily detected in normal mammalian cells, and it is found at elevated levels in cancer cells (7, 8, 9, 10, 11, 12, 13, 14). While oxoA does not appear to be strongly mutagenic in *Escherichia coli*, based on cellular and *in vitro* assays (Klenow fragment of DNA pol I) (15, 16, 17, 18), it is mutagenic in mammalian cells, causing A→C transversions (predominantly) and A→G transitions (16, 19). Mammalian DNA polymerases, including pol α, pol β, and pol η, incorporate dGTP opposite oxoA (template), yielding mispairs that can cause A→C transversions (15, 16, 18, 20, 21, 22). Pol β also incorporates dCTP opposite oxoA, which can cause A→G transitions (16, 23). An archaeal DNA polymerase, Dpo4 (*Saccharolobus solfataricus*), incorporates dGTP opposite oxoA and does so with higher efficiency than incorporation of dATP opposite oxoG (24). Interestingly, several reverse transcriptases incorporate dGTP opposite 8-oxo-rA (RNA template) (25). Thus, replication past oxoA is mutagenic for a broad range of polymerases.

**Figure 1:**
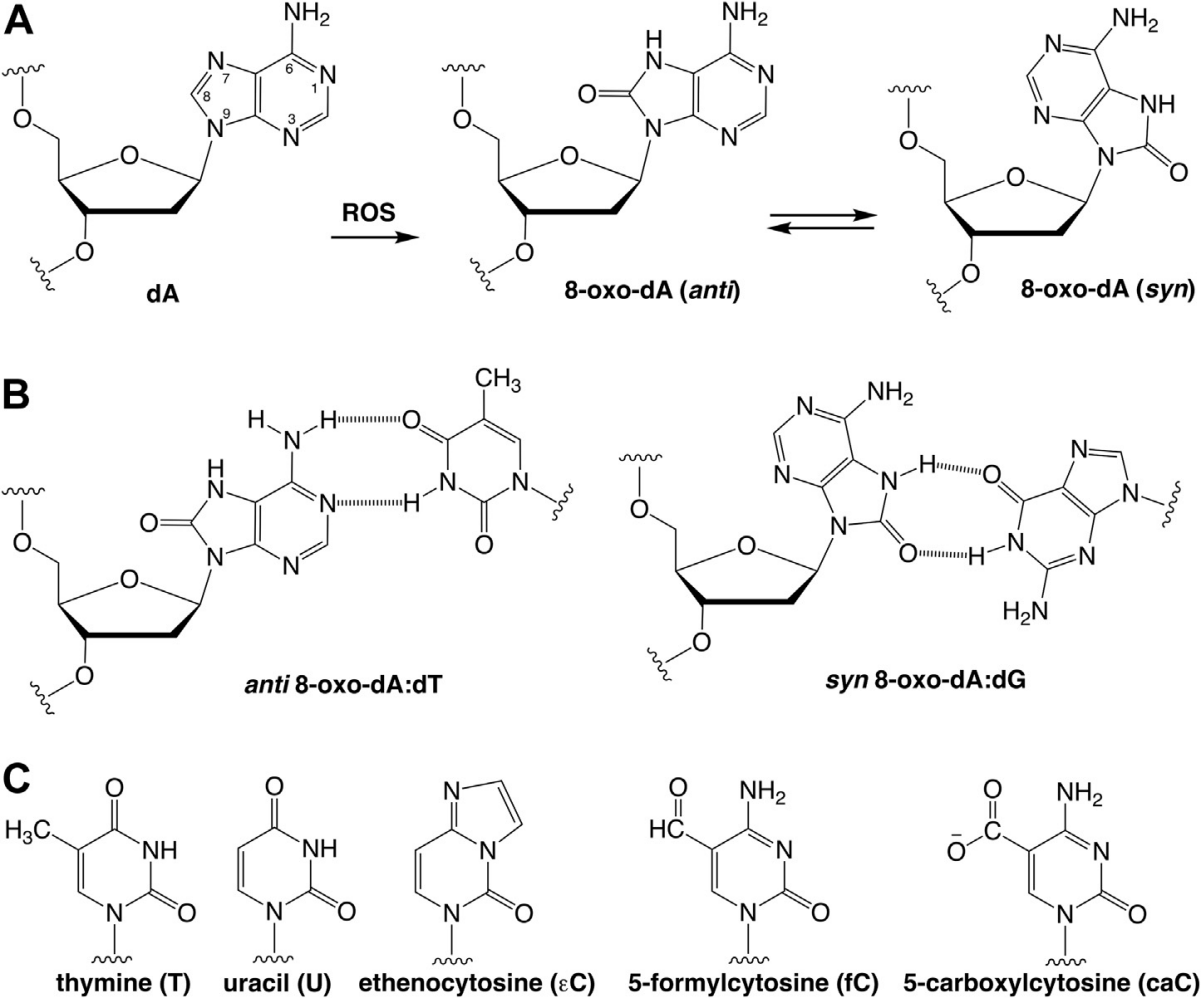
**Structure of 7,8-dihydro-8-oxoadenosine (8-oxo-dA), base pairs with dT and dG, and pyrimidine bases considered in this work.***A*, oxidation of dA gives 8-oxo-dA, which can adopt *anti* and *syn* conformations; steric hindrance involving the 8-oxygen, observed for *anti*, is relieved for *syn* 8-oxo-dA. *B*, 8-oxo-dA (*anti*) forms a canonical base pair with dT (similar to dA:dT), whereas 8-oxo-dA, in the *syn* conformation, forms a wobble pair with dG, in free DNA. *C*, some of the established pyrimidine substrates for TDG and MUG. MUG, mismatch uracil glycosylase; TDG, thymine DNA glycosylase.

A crystal structure of DNA containing G⋅oxoA pairs shows the oxoA nucleotide adopts a syn conformation and forms a wobble pair with dG (*anti*) (Fig. 1*B*) (26). Recent crystal structures reveal how human pol β and pol η generate mutagenic G⋅oxoA pairs. In the pol β active site, the 8-oxo-dA nucleotide (template) adopts the *syn* conformation and forms a Hoogsteen base pair with the incoming dGTP that features three hydrogen bonds and Watson–Crick-like geometry (21). By contrast, pol β insertion of dTTP involves *anti*-oxoA (template) and canonical Watson–Crick base pairing geometry (Fig. 1*B*). Pol η exhibits the same result for dTTP incorporation opposite oxoA, whereas incorporation of dGTP involves *syn*-oxoA with wobble base pairing stabilized by the enzyme (22). A structure of the archaeal polymerase Dpo4 shows that a template *syn*-oxoA forms a Hoogsteen pair with dGTP (24). Consistent with these structural observations, thermal melting studies of duplex DNA indicate that oxoA pairs are more stable for guanine relative to the three other bases, with melting temperatures (*T*_m_) trending as G⋅oxoA > T⋅oxoA > C⋅oxoA > A⋅oxoA (18). Notably, equivalent base-pairing stability was observed for G⋅oxoA and a canonical A⋅T pair.

The oxoA lesion could potentially arise in DNA through incorporation of the oxidized nucleotide, 8-oxo-dATP. Notably, the nucleotide pool is far more susceptible to oxidation than are the bases in DNA (27). Although DNA polymerase incorporation of 8-oxo-dATP has not been reported (to our knowledge), incorporation of 8-oxo-dGTP has been observed for many human polymerases, including pol β, pol η, and pol κ (27). The potential for 8-oxo-dATP incorporation is supported by findings that human enzymes that sanitize the nucleotide pool are known to deplete 8-oxo-dATP and 8-oxo-dADP. Indeed, MTH1 (MutT homolog 1) exhibits similar efficiency for hydrolyzing 8-oxo-dATP and 8-oxo-dGTP (28). By contrast, *E. coli* MutT acts only on 8-oxo-dGTP, consistent with the findings that 8-oxoA is not highly mutagenic in *E. coli*. In addition, human NUDT5 (ADP-sugar pyrophosphatase), which hydrolyzes nucleoside diphosphates, acts with similar efficiency on 8-oxo-dADP and 8-oxo-dGDP (29). These findings support the possibility that human DNA polymerases incorporate 8-oxo-dATP into DNA.

The presence of oxoA in DNA poses threats in addition to mutagenesis. For example, oxoA perturbs the exonuclease activity of the WRN (bifunctional 3′-5′ exonuclease/ATP-dependent helicase) protein (30, 31) and hinders repair activity at a proximal abasic site by apurinic/apyrimidimic endonuclease 1 and bifunctional DNA glycosylases (32). RNA polymerase II is halted by oxoA and oxoG, and the oxoA blockage is not alleviated by the transcription elongation factor TFIIS (33). In addition, oxoA, but not oxoG, can form interstrand crosslinks with A or G under oxidative conditions *in vitro* (34). These findings underscore the importance of repairing oxoA lesions in DNA.

Multiple studies have reported oxoA excision activity in human cells (9, 23). Two DNA glycosylases that act preferentially on other lesions, OGG1 (8-oxoguanine DNA glycosylase) and NEIL1 (endonuclease VIII like protein 1) can excise oxoA but only from C⋅oxoA pairs and with modest activity (23, 35, 36). Another study reported that human thymine DNA glycosylase (TDG) excises oxoA, with similar activity for base pairs with G, T, or C but with no detectable activity for A⋅oxoA pairs (37). This and another study reports activity for oxoA excision in extracts of murine embryonic fibroblasts but not TDG-deficient extracts (38).

We were highly intrigued by reports that TDG excises oxoA, given that its established biological substrates are all modified pyrimidines (paired with G) including T, U, 3,*N*^4^-ethenocytosine (εC), 5-formylcytosine (fC), and 5-carboxylcytosine (caC) (39, 40, 41, 42, 43). We sought to rigorously characterize this activity by performing single-turnover kinetic assays for TDG acting on oxoA and the known pyrimidine substrates. We also investigated the effect of the opposing base on oxoA activity, given that polymerases can generate G⋅oxoA and C⋅oxoA mispairs, whereas T⋅oxoA pairs are likely generated by oxidation of adenine bases in DNA. We also asked whether the N- and C-terminal intrinsically disordered regions of TDG, which flank its ordered catalytic domain, play a substantial role in excising oxoA. In addition, we interrogated the role of two conserved catalytic residues (N140 and N191). Finally, we characterized the activity of a bacterial ortholog, *E. coli* mismatch uracil glycosylase (MUG), which features 32% amino acid sequence identity to TDG but has different substrate specificity.

## Results

### Experimental considerations

**Figure 2:**
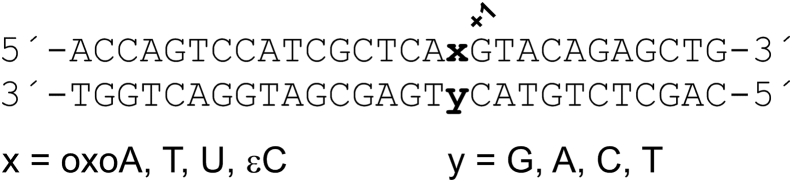
**Construct and nomenclature for the 28 bp DNA substrates used in this study.** The substrate nomenclature used herein is “y·x,” where x represents the target base and y the opposing base. For example, the “G·oxoA” substrate indicates that oxoA is the target base (x) and G is the opposing base (y). The “+1” indicates the base positioned immediately 3′ of the target base. The identity of the +1 base can have a strong effect on TDG activity for some substrates (see main text). TDG, thymine DNA glycosylase.

The 28 bp duplex DNA substrates used in this study shared the same basic construct, as shown in Figure 2. The upper (“target”) strand contained oxoA, T, U, or εC at the target site (“x”). Previous studies have shown that TDG activity can depend sharply on the base positioned immediately 3′ of the target site, with optimal activity for guanine at the 3′ (or +1) position, not only for excision of thymine from G·T mispairs but also for excision of uracil and some 5-substituted uracils (44, 45, 46). These findings reflect the strong specificity of TDG for excising deaminated 5-methylcytosine (thymine) from a CpG context. As such, the DNA construct used in this work contains guanine at the +1 site (Fig. 2). The complementary strand contained one of the four canonical bases at the “y” site, which is positioned to form a base pair with the x site of the target strand.

Because the catalytic turnover of TDG and MUG is severely limited by slow release of reaction product, apurinic/apyrimidinic DNA, and by product inhibition (47, 48), the rate constant obtained from steady-state kinetics experiments (*k*_cat_) is not useful for comparing activity among various enzymes or substrates. Rather, glycosylase activity was monitored using single-turnover kinetics experiments performed under saturating enzyme conditions (49). The resulting rate constants (*k*_obs_) are not impacted by enzyme–substrate association or by product release or product inhibition and thereby approximate the maximal rate of product formation (*k*_obs_ ≈ *k*_max_) (49). The observed rate constants report on steps including the chemical step and conformational changes that occur after enzyme–substrate association but prior to chemistry, including nucleotide flipping.

### TDG rapidly excises 8-oxoadenine from DNA

**Figure 3:**
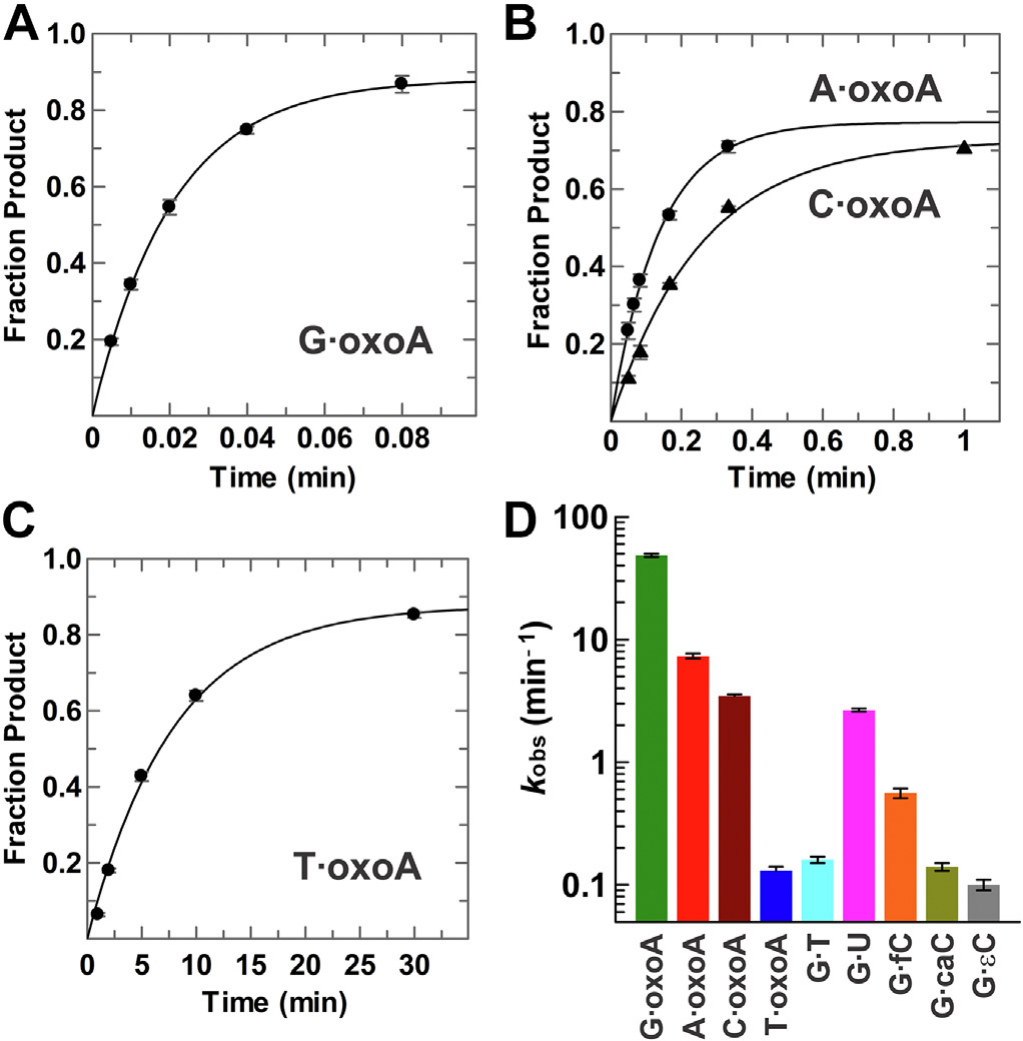
**TDG exhibits much higher activity for excising oxoA compared with its established pyrimidine substrates.***A*, TDG (2.5 μM) excises oxoA from a G·oxoA substrate with *k*_obs_ = 48.4 ± 1.6 min^−1^. *B*, TDG excises oxoA from A·oxoA pairs with *k*_obs_ = 7.33 ± 0.33 min^−1^ (*circles*) and from C·oxoA pairs with *k*_obs_ = 3.46 ± 0.12 min^−1^ (*triangles*). *C*, TDG removes oxoA from T·oxoA pairs with *k*_obs_ = 0.13 ± 0.01 min^−1^. *D*, comparison of TDG activity (*k*_obs_) for the four oxoA base pairs and the established pyrimidine substrates; *k*_obs_ is shown in log format with standard deviation. The data for G·fC and G·caC were previously reported (see main text). oxoA, 7,8-dihydro-8-oxoadenine; TDG, thymine DNA glycosylase.

Previous studies show that TDG excision of uracil, thymine, and other 5-substituted uracils (*e.g.*, 5-fluorouracil) is highly dependent on the opposing base, where excision is much faster when these bases are paired with guanine rather than adenine (45). As such, we first examined TDG activity for excision of oxoA from a G·oxoA pair in DNA. Remarkably, we find that TDG exhibits extraordinarily high activity for G·oxoA pairs, with *k*_obs_ = 48.4 ± 1.6 min^−1^ (Fig. 3*A* and Table 1) and a reaction half-life of under 1 s. To provide direct points of comparison, we determined TDG activity for G·T, G·U, and G·εC pairs in the same DNA construct (Fig. S1 and Table 1), and we obtained *k*_obs_ values in excellent agreement with previous observations (44, 50). Our results reveal that TDG activity is vastly higher for G·oxoA relative to pyrimidine substrates, with differences of 303-, 18-, and 484-fold for G·T, G·U, and G·εC, respectively (Table 1 and Fig. 3*D*). Moreover, our previous studies indicate activity differences of 86- and 346-fold for G·fC and G·caC, respectively, relative to G·oxoA (51, 52, 53). The highly rapid excision of oxoA is remarkable given that it is substantially bulkier than the established pyrimidine substrates and that TDG has not previously been reported to efficiently remove other purine bases. Notably, the activity (*k*_obs_) reported here for TDG processing of a G·oxoA substrate is 30-fold higher than a previously reported value (*k*_max_ = 1.6 min^−1^) (37).

**Table 1:** Glycosylase activity

Enzyme	Substrate	*k*_obs_ (min^−1^)	Relative to G·oxoA	Relative to TDG (or TDG^82-308^)
TDG	G·oxoA	48.4 ± 1.6		
	A·oxoA	7.33 ± 0.33	1/7	
	C·oxoA	3.46 ± 0.12	1/14	
	T·oxoA	0.13 ± 0.01	1/372	
	G·T	0.16 ± 0.01	1/303	
	G·U	2.66 ± 0.08	1/18	
	G·εC	0.10 ± 0.01	1/484	
	G·fC^a^	0.56 ± 0.05	1/86	
	G·caC^b^	0.14 ± 0.01	1/346	
TDG^82–308^	G·oxoA	56.7 ± 2.5		1.2
	A·oxoA	12.6 ± 1.0	1/5	1.7
	C·oxoA	4.21 ± 0.22	1/13	1.2
	T·oxoA	0.17 ± 0.01	1/326	1.3
	G·T	0.22 ± 0.01	1/263	1.4
	G·U	3.15 ± 0.06	1/18	1.2
TDG^111–308^	G·oxoA	36.7 ± 1.2		1/1.3
	A·oxoA	2.08 ± 0.07	1/18	1/3.5
	C·oxoA	1.58 ± 0.05	1/23	1/2.2
	T·oxoA	0.029 ± 0.001	1/1266	1/4.5
	G·T	0.039 ± 0.001	1/941	1/4.1
	G·U	1.26 ± 0.03	1/29	1/2.1
N191A-TDG^82-308^	G·oxoA	0.14 ± 0.01		1/394
	A·oxoA	0.060 ± 0.002	1/2.4	1/210
	C·oxoA	0.062 ± 0.002	1/2.3	1/68
	T·oxoA	0.0043 ± 0.0006	1/33	1/40
	G·T	0.022 ± 0.001	1/7	1/10
	G·U	0.53 ± 0.04	3.7	1/6
N140A-TDG^82-308^	G·oxoA	0.0050 ± 0.0001		1/11,340
	A·oxoA	(5.8 ± 0.2) × 10^−4^	1/8.6	1/21,728
	C·oxoA	(2.3 ± 0.1) × 10^−4^	1/22	1/18,711
	T·oxoA	(5.9 ± 0.4) × 10^−6^	1/847	1/29,492
MUG	G·oxoA	(3.6 ± 0.1) × 10^−4^		
	A·oxoA	(1.3 ± 0.1) × 10^−4^	1/2.7	
	C·oxoA	(2.7 ± 0.1) × 10^−4^	1/1.3	
	T·oxoA^c^	<2.3 × 10^−6^	<1/155	
	G·U	0.97 ± 0.03	2725	
	G·εC	10.6 ± 0.7	29,775	

The *k*_obs_ values represent the average (±standard error) obtained from fitting the progress curves for at least three independent reactions to Equation 1.

a *k*_obs_ was previously determined under similar experimental conditions (52).

b *k*_obs_ was previously determined under similar experimental conditions (42).

c Activity not detected; *k*_obs_ represents upper limit value.

### TDG excises oxoA paired with any DNA base

We also characterized TDG activity for excising oxoA paired with the three other canonical DNA bases (A, C, and T). We find that TDG exhibits high activity for excising oxoA from A·oxoA pairs, with *k*_obs_ = 7.33 ± 0.33 min^−1^ (Fig. 3*B*), which is sevenfold lower than for G·oxoA pairs but still much higher than for the established pyrimidine substrates. This is quite remarkable in light of previous findings that TDG has exceedingly slow or no detectable activity for excising thymine, uracil, or 5-substituted uracils when these bases are paired with adenine (45). Evidently, mechanisms that preclude excision of thymine from A·T pairs are relaxed for excision of oxoA paired with adenine. Notably, a recent study finds that TDG excision of fC and caC is similarly efficient when these bases are paired with adenine or guanine (54).

TDG also exhibits high activity for excising oxoA from C·oxoA pairs, with *k*_obs_ = 3.46 ± 0.12 min^−1^ (Fig. 3*B*), which still exceeds that observed for the pyrimidine substrates. Remarkably, oxoA activity is sharply reduced for T·oxoA pairs; *k*_obs_ = 0.13 ± 0.01 min^−1^ (Fig. 3*C*). Indeed, excision activity is reduced 372-fold for oxoA paired with T *versus* G. Our findings show that oxoA excision exhibits the following trend in *k*_obs_ with regard to the opposing base: G > A > C >> T (Fig. 3*D*). Our results are in sharp contrast with the report that TDG does not excise oxoA from A·oxoA pairs and that it exhibits nearly equivalent activity for excising oxoA paired with T, C, or G (37). However, our observed activity (*k*_obs_) for TDG acting on the T·oxoA substrate is similar to the reported value (0.35 min^−1^) and supports the conclusion that TDG activity for T·oxoA is comparable to that for some pyrimidine substrates (G·T, G·εC, and G·caC) (37).

We find no evidence that TDG excises thymine from the T·oxoA substrate. This could potentially reflect a DNA context effect, given previous findings that excision of thymine (from G·T) depends strongly on the 3′-neighboring base. In particular, thymine excision is greatly suppressed if its 3′-neighbor is thymine rather than guanine (46, 55). Such is the context for the T paired with oxoA in the substrate (T·oxoA) used for these studies (Fig. 2, base “y”). A previous study observed that excision of T from a T·oxoA substrate is extremely low even for a substrate in which the T was in its most favorable sequence context (with G as the 3′-neighbor) (38). Additional studies will be needed to characterize the context dependence of thymine excision from T·oxoA pairs by TDG.

### Disordered regions of TDG are not essential for oxoA activity

**Figure 4:**
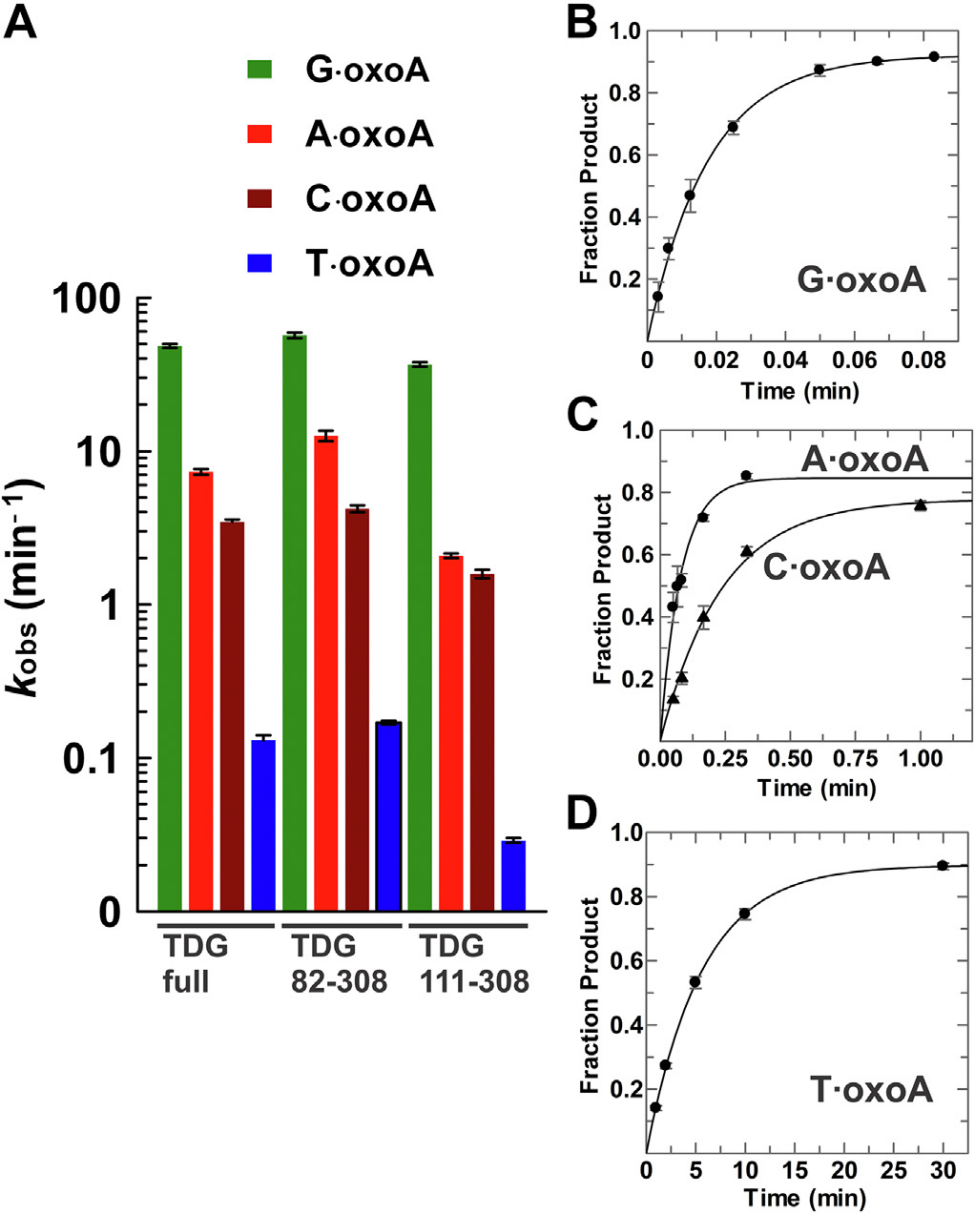
**Disordered regions of TDG are not essential for oxoA excision.***A*, activity of TDG, TDG^82–308^, and TDG^111–308^ for the four oxoA substrates (*k*_obs_ in log scale). TDG^82–308^ (2.5 μM) processes (*B*) the G·oxoA substrate with *k*_obs_ = 56.7 ± 2.5 min^−1^, the A·oxoA substrate (*C*, *circles*) with *k*_obs_ = 12.6 ± 1.0 min^−1^, the C·oxoA substrate (*C*, *triangles*) with *k*_obs_ = 4.21 ± 0.22 min^−1^, and (*D*) removes oxoA from the T·oxoA substrate with *k*_obs_ = 0.17 ± 0.01 min^−1^. oxoA, 7,8-dihydro-8-oxoadenine; TDG, thymine DNA glycosylase.

Human TDG (410 residues) comprises a structured catalytic domain of ∼190 residues flanked by N- and C-terminal regions of nearly equivalent length that are both intrinsically disordered, as indicted by NMR (51, 56). We sought to define the role of the disordered regions in oxoA excision. Our studies included TDG^82–308^, a construct that exhibits substrate binding and glycosylase activity equivalent to full-length TDG for G·T and G·fC pairs (51, 52). Crystal structures of DNA-bound TDG^82–308^ show that the structured region includes residues 108 to 302 (51, 53). NMR studies of DNA-bound TDG^82–308^ indicate that residues 82 to 110 are disordered even though they contribute to substrate binding and catalysis (for G·T pairs) (51). Notably, oxoA excision activity is modestly elevated (1.2- to 1.7-fold) for TDG^82–308^ relative to TDG (Fig. 4 and Table 1). Similarly, activity on the pyrimidine substrates (G·T, G·U, and G·εC) is slightly elevated for TDG^82–308^ relative to TDG (Fig. S2).

Using the same oxoA and pyrimidine substrates, we determined the activity of TDG^111–308^ (aka TDG^cat^), a minimal catalytic construct that lacks the 29 disordered N-terminal residues of TDG^82–308^ (Fig. S3 and Table 1). Activity for the oxoA substrates is modestly reduced (1.3- to 4.5-fold) for TDG^111–308^ relative to TDG (Fig. 4*A*). Activity for G·T and G·U is also modestly reduced for TDG^111–308^ *versus* TDG (Fig. S3), in agreement with previous observations (47, 51). Together, our results indicate that the disordered regions of TDG are not essential for activity on oxoA substrates, similar to findings for pyrimidine substrates, although residues 82 to 110 of TDG^82–308^ enhance its activity relative to that of TDG^111–308^. Our findings do not support the suggestion that the N-terminal region of TDG is essential for oxoA excision (37). This discrepancy could reflect experimental conditions, as the prior studies were performed with a 20-fold lower concentration of TDG^111–308^ at a temperature (37 °C) that might destabilize the enzyme (51).

### N191 is critical for efficient oxoA excision

**Figure 5:**
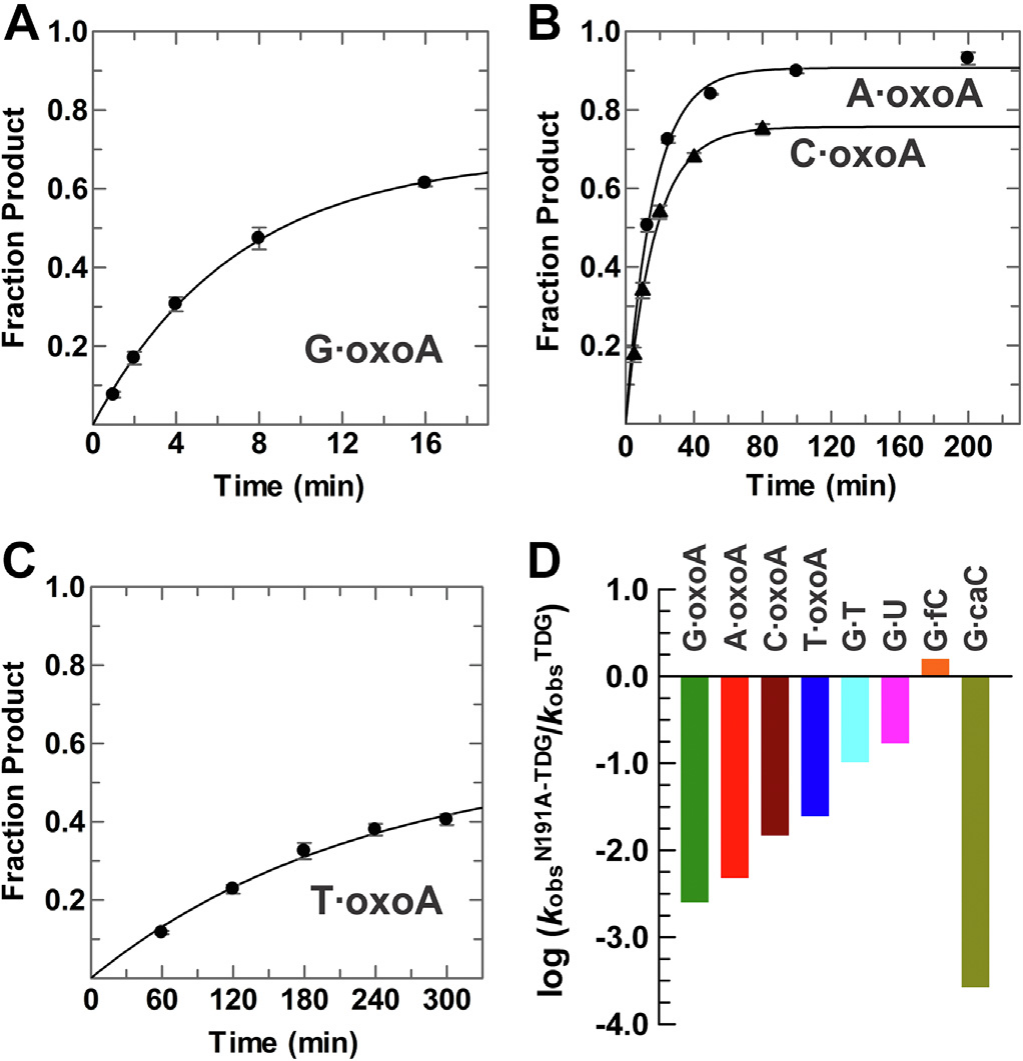
**The N191A mutation causes large reductions in oxoA activity.** N191A-TDG^82–308^ (5 μM) processes DNA substrates containing a G·oxoA pair with *k*_obs_ = 0.14 ± 0.01 min^−1^ (*A*), an A·oxoA pair with *k*_obs_ = 0.060 ± 0.002 min^−1^ (*B*, *circles*), a C·oxoA pair with *k*_obs_ = 0.062 ± 0.002 min^−1^ (*B*, *triangles*), and a T·oxoA pair with *k*_obs_ = 0.0043 ± 0.0006 min^−1^ (*C*). A *bar graph* (*D*) shows the impact of the N191A mutation on TDG activity (*k*_obs_, log scale) for oxoA and pyrimidine substrates. Effects for G·fC and G·caC are based on our previous studies (main text). oxoA, 7,8-dihydro-8-oxoadenine; TDG, thymine DNA glycosylase.

We also sought to characterize the role in oxoA excision for an active site residue of TDG that mediates its activity for some of the established pyrimidine substrates. Asn191 is a strictly conserved residue of TDG that is essential for caC excision, dispensable for fC excision, and contributes moderately to G·T and G·U activity (50, 53, 57). Remarkably, the N191A mutation causes a large 394-fold loss in activity for G·oxoA pairs (Fig. 5*A* and Table 1). The mutation also strongly hinders activity for the three other oxoA substrates, with reductions in *k*_obs_ of 210-, 68-, and 40-fold for A·oxoA, C·oxoA, and T·oxoA pairs, respectively (Fig. 5). By comparison, the N191A mutation causes modest 10- and 6-fold losses in activity for G·T and G·U substrates (Fig. S4) (50). Notably, the damaging effect of this mutation on oxoA activity, which is exceeded only by the 3800-fold effect on caC activity (53), points to a major catalytic role for N191 in oxoA excision.

### N140 is essential for oxoA excision

**Figure 6:**
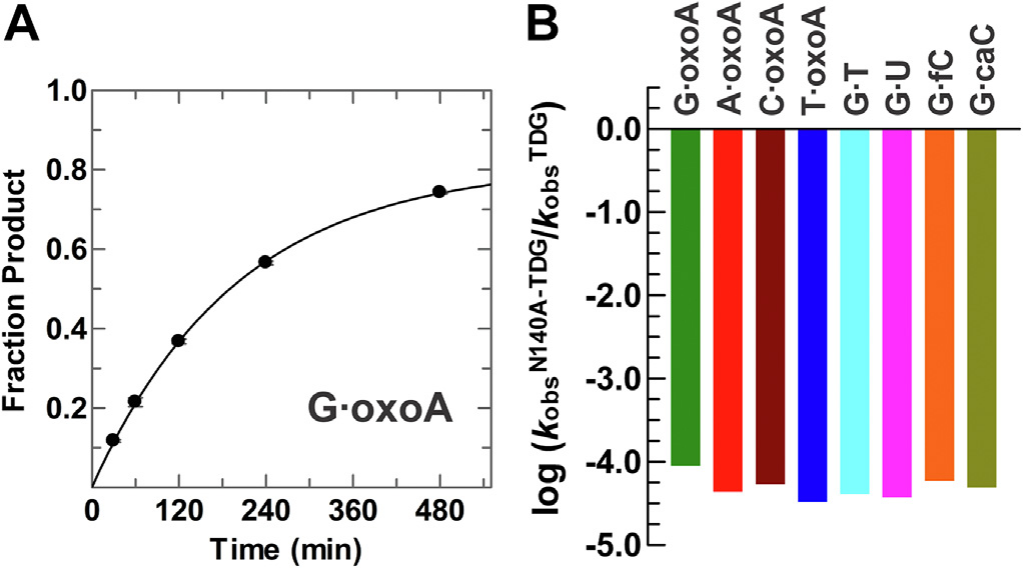
**The N140A mutation in TDG causes massive reductions in activity for oxoA and pyrimidine substrates.***A*, N140A-TDG^82–308^ (2.5 μM) processes G·oxoA pairs with *k*_obs_ = 0.0050 ± 0.0001 min^−1^. *B*, effect of the N140A mutation on TDG^82–308^ activity for oxoA and pyrimidine substrates. The effects for pyrimidine substrates are from our previous studies (main text). oxoA, 7,8-dihydro-8-oxoadenine; TDG, thymine DNA glycosylase.

A series of high-resolution crystal structures show that N140, a strictly conserved residue in the TDG–MUG enzyme family, serves to bind and position the nucleophilic water molecule in the enzyme–substrate complex (51, 52, 53). Consistent with such a central catalytic role, the N140A mutation causes a massive loss in TDG activity (>10^4^-fold) for pyrimidine substrates (52, 53, 58). Here, we show that the N140A mutation exerts similarly large effects on oxoA activity with *k*_obs_ decreases ranging from 11,300-fold (G·oxoA) to 29,500-fold (T·oxoA) (Table 1, Figs. 6 and S5). These results indicate an essential catalytic role for N140 in oxoA excision, likely the same role indicated by the studies noted previously for the pyrimidine substrates.

### Bacterial MUG lacks significant oxoA activity

**Figure 7:**
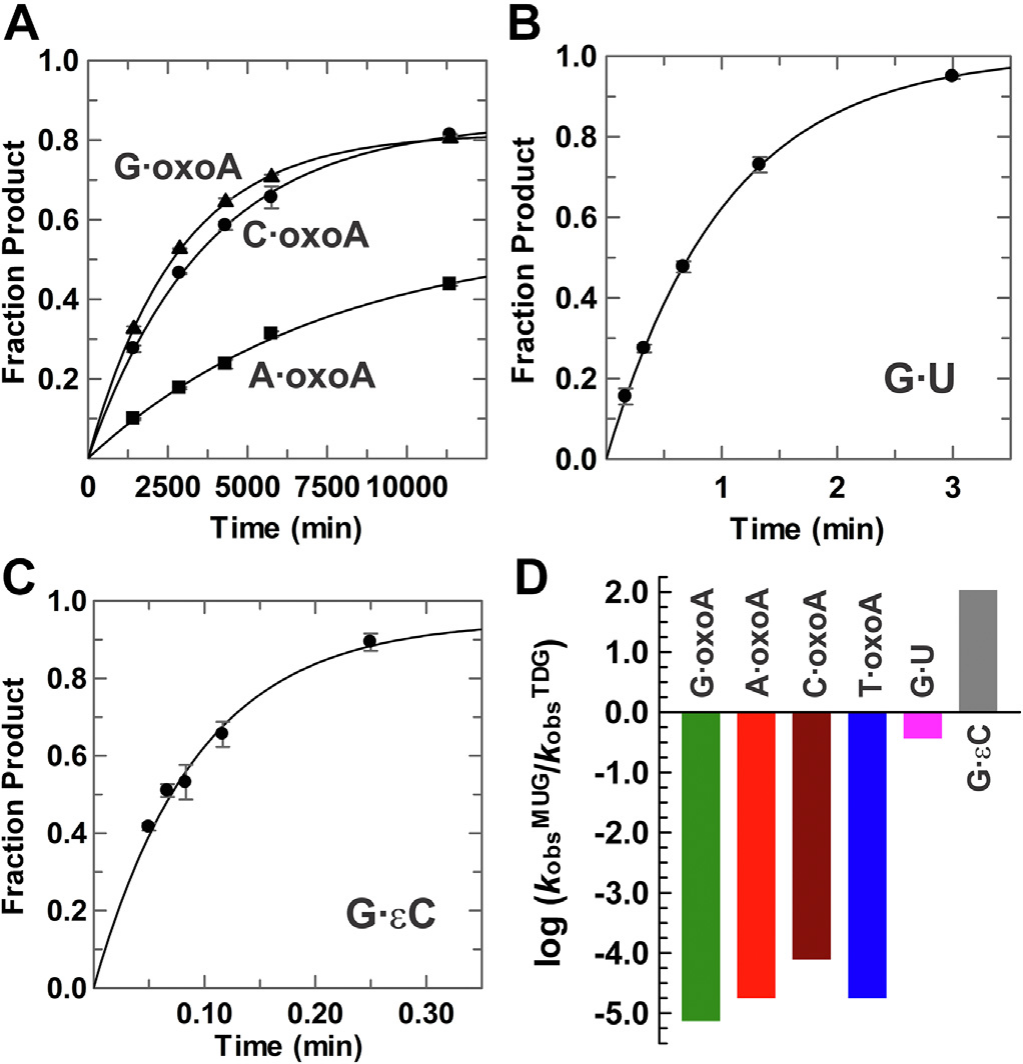
**Excision of oxoA, U, and εC by *Escherichia coli* MUG.***A*, MUG excises oxoA from G⋅oxoA pairs with *k*_obs_ = (3.6 ± 0.1) × 10^−4^ min^−1^ (*triangles*), from A⋅oxoA pairs with *k*_obs_ = (1.3 ± 0.1) × 10^−4^ min^−1^ (*squares*), and from C⋅oxoA pairs with *k*_obs_ = (2.7 ± 0.1) × 10^−4^ min^−1^ (*circles*). *B*, MUG excises U from G⋅U pairs with *k*_obs_ = 0.97 ± 0.03 min^−1^. *C*, MUG removes εC from G·εC pairs with *k*_obs_ = 10.6 ± 0.7 min^−1^. *D*, activity of MUG relative to TDG for oxoA and pyrimidine substrates (activity ratio in log format). The activity difference for T⋅oxoA represents a minimum since MUG exhibited no detectable activity for this substrate. εC, 3,*N*^4^-ethenocytosine; MUG, mismatch uracil glycosylase; oxoA, 7,8-dihydro-8-oxoadenine.

Given that *E. coli* MUG exhibits 32% amino acid sequence identity to human TDG (residues 123–298) (59) and that the enzymes exhibit some substrate overlap, albeit with varying efficiency, we sought to examine MUG activity for excising oxoA. We find that MUG exhibits exceedingly slow activity for removing oxoA from G⋅oxoA pairs; *k*_obs_ = 0.00036 ± 0.00001 min^−1^ (Fig. 7*A*). MUG exhibits similarly low activity for A⋅oxoA and C⋅oxoA pairs (Fig. 7*A*), and it has no detectible activity for T⋅oxoA pairs (over a period of 72 h; not shown). Thus, MUG lacks significant activity for oxoA excision, regardless of base pairing, in contrast with a previous report (37).

Our results reveal a vast difference in the activity of TDG and MUG for oxoA excision, where the activity ratio is 136,000 for G⋅oxoA, 56,000 for A⋅oxoA, and 13,000 for C⋅oxoA pairs. To provide additional context, we determined MUG activity for two established substrates, finding *k*_obs_ = 0.97 ± 0.03 min^−1^ for G·U (Fig. 7*B*) and *k*_obs_ = 10.6 ± 0.8 min^−1^ for G·εC (Fig. 7*C*). The latter results are in agreement with a study that employed similar single-turnover conditions (48). While MUG and TDG exhibit a modest threefold difference in activity for G·U, MUG possesses 100-fold higher activity for G·εC (Fig. 7*D*). The divergence in specificity of these orthologs is underscored by comparing the activity ratio for G·oxoA and G·εC substrates (*k*_obs_^G·oxoA^/*k*_obs_^G·εC^), which reveals a difference of 14 million.

## Discussion

Our results reveal strikingly high activity for excision of oxoA by TDG, particularly for G⋅oxoA mispairs. Indeed, activity is 18- to 480-fold higher for G⋅oxoA relative to the established pyrimidine substrates of TDG (G⋅U, G⋅T, G⋅fC, G⋅caC, and G⋅εC). TDG activity for A⋅oxoA and C⋅oxoA mispairs is also high, exceeding that for the pyrimidine substrates. The oxoA activity is sharply reduced for T⋅oxoA pairs and is comparable to the pyrimidine substrates. We performed the activity assays at room temperature (23 °C) because TDG and MUG are not sufficiently stable at physiological temperature (37 °C) to perform the full suite of experiments conducted in these studies, including some of very long duration (several days) (48, 51, 60). Nevertheless, previous studies of TDG with other substrates suggest that its oxoA activity could be about twofold higher at 37 °C (46, 60).

The robust oxoA excision activity observed here could be important for avoiding mutations or could possibly play a role in causing them, depending on how the oxidized adenine arises in DNA. For example, if T⋅oxoA pairs arise through oxidation of DNA, then TDG excision of oxoA and followed by base excision repair to give a T⋅A pair could help to avoid mutations. This activity could also be beneficial for handling T⋅oxoA pairs generated by incorporation of 8-oxo-dATP by a DNA polymerase. Moreover, excision of oxoA would likely be beneficial if a polymerase incorporates 8-oxo-dATP opposite one of the other three canonical bases (A, G, or C). While we are not aware of any reported studies that investigated polymerase incorporation of 8-oxo-dATP, previous studies show that several human DNA polymerases (pol β, pol η, and pol κ) incorporate 8-oxo-dGTP into DNA (27). As noted previously, the possibility that polymerases incorporate 8-oxo-dATP is supported by findings that two human nucleotide-sanitizing enzymes of the NUDIX family, HMTH1 and NUDT5, have been shown to deplete 8-oxo-dATP and 8-oxo-dADP from the nucleotide pool (28, 29). These observations, together with the oxoA excision activity of TDG, indicate that additional studies are warranted to characterize the potential incorporation of 8-oxo-dATP into DNA by mammalian DNA polymerases. As noted previously, studies of mammalian DNA polymerases (pol β, pol η, etc.) indicate they preferentially incorporate dTTP opposite oxoA (template), but they can also incorporate dGTP or dCTP, generating G⋅oxoA or C⋅oxoA pairs. TDG excision of oxoA from these mispairs could be mutagenic because repair would occur on the template (oxoA) rather than the misincorporated base (G or C). The same argument would apply to TDG action on an A⋅oxoA pair generated by incorporation of dATP opposite oxoA, though such activity by a polymerase has not yet been reported (to our knowledge).

It is of interest to compare our findings for oxoA excision by TDG and MUG with those reported previously by Saparbaev *et al.* (37). We find that TDG activity for a G·oxoA substrate is 30-fold faster than the previous report (*k*_max_ = 1.6 min^−1^), and we observe robust activity for A·oxoA while the prior study found that TDG lacks detectible A·oxoA activity. We also observe relatively high TDG activity for C·oxoA, about 10-fold higher than previously reported (*k*_max_ = 0.32 min^−1^). We find that oxoA activity depends strongly on the opposing base, whereas the prior study found similar activity for oxoA paired with G, C, or T. On the other hand, our observed TDG activity for T·oxoA is similar to the previous finding and supports the conclusion that activity for this substrate is comparable to that for some pyrimidine substrates of TDG, including G·T, G·εC, and G·caC. We do not find an essential role for disordered N-terminal residues in processing oxoA substrates, in contrast with the conclusion that the disordered N-terminal region is essential for oxoA excision, based on the observation that TDG^111–308^ lacked activity for a T·oxoA substrate (37). We find that TDG^111–308^ can process T·oxoA and the three other oxoA pairs, albeit with somewhat reduced activity relative to full-length TDG. Finally, we find that *E. coli* MUG lacks significant oxoA excision activity, with rate constants that are 4 to 5 orders of magnitude below those observed for TDG, whereas the prior study found that MUG has substantial oxoA activity. The prior study found that MUG processes T·oxoA substrates with *k*_max_ = 0.085 min^−1^, whereas we find no detectible MUG activity for T·oxoA. While the immediate DNA context for oxoA in the substrates differed between the two studies, with a -TCAxGTA- context (x = oxoA) for our 28 bp substrates and a -CGCxCGC- context for their 40 bp substrates, this seems unlikely to account for the differing results, at least for TDG. Indeed, the prior study found that activity for a T·oxoA substrate was not strongly dependent on the base positioned immediately 3′ or 5′ of oxoA (37). The buffers used for the activity assays were similar (theirs comprising 20 mM Tris–HCl [pH 8.0], 100 mM NaCl, 1 mM DTT, and 0.1 mg/ml bovine serum albumin), and this also seems unlikely to account for the differential findings. The discrepancies could potentially reflect differences in experimental temperature and enzyme concentration. We used a temperature of 23 °C, for which the enzymes (TDG, TDG^111–308^, and MUG) are more stable than at 37 °C (48, 51, 60), the temperature used in the previous work (37). The prior study was performed using enzyme and substrate concentrations of 0.50 and 0.05 μM, respectively, whereas we used a 10-fold higher concentration of substrate (0.50 μM) and 5- to 20-fold higher concentrations of enzyme (2.5–10 μM). The enzymes are likely to be more stable under our experimental conditions of lower temperature and higher concentrations of enzyme and substrate (48, 51, 60). While MUG is more stable at lower temperature, it is possible that it exhibits weak T·oxoA activity at 37 °C but not at 23 °C. Additional studies would be needed to determine whether these or other explanations account for the differences in our results and those of the prior study (37).

### Implications for the mechanism of oxoA excision by TDG

Our results reveal that TDG excision of oxoA depends substantially on the opposing base, with the following trend in activity (*k*_obs_): G⋅oxoA > A⋅oxoA > C⋅oxoA >> T⋅oxoA. Moreover, the span in activity is remarkably large, with a nearly 400-fold difference between G⋅oxoA and T⋅oxoA. Notably, results from previous thermal melting of DNA containing the oxoA pairs suggest the TDG activity trend is not explained simply by differences in the stability of oxoA pairs, given the following trends in melting transition temperature (*T*_m_): G⋅oxoA > T⋅oxoA > C⋅oxoA > A⋅oxoA (18). If base pair stability were the major determinant of TDG activity for the oxoA pairs, one would anticipate activity to increase as *T*_m_ decreases. By contrast, TDG activity is greatest for the most stable pair (G⋅oxoA). Together, these results suggest a mechanism for preferential excision of oxoA paired with guanine, despite the need to disrupt a relatively strong base pair (equivalent to that of a normal A⋅T pair) (18). The results also suggest a mechanism that reduces activity for oxoA paired with thymine. The strong influence of the opposing base on oxoA excision is interesting and worthy of additional mechanistic investigation.

Our findings indicate a critical role for N191 in oxoA excision by TDG. Indeed, the N191A mutation reduces activity by 40- to 400-fold for the oxoA pairs, with the largest effect for G⋅oxoA. Notably, this mutation has a much smaller effect for G⋅U and G⋅T substrates (6- and 10-fold) and does not significantly alter TDG activity for G⋅fC pairs (50, 57). The observations here for oxoA are reminiscent of findings for G⋅caC, where the N191A mutation reduces activity by 3800-fold (53, 57). The large mutational effect for oxoA activity could reflect a role for N191 in stabilizing the flipped conformation of the 8-oxo-dA nucleotide and/or an effect on the chemical step, which includes *N*-glycosyl bond cleavage and nucleophile (water) addition. Previous studies indicate that for most pyrimidine substrates, TDG activates the leaving group (nucleobase) through electrostatic interactions to stabilize an anionic form of the departing base (61, 62). However, unlike other pyrimidines, TDG excision of caC involves activation of the leaving group *via* protonation (acid catalysis). The large N191A mutational effect on caC activity, together with biochemical and structural observations, suggests that N191 plays a role in facilitating acid-catalyzed caC excision (53, 57, 60). Additional studies are needed to discern whether TDG excision of oxoA is acid catalyzed, and if so whether this involves N191. Notably, previous studies indicate that 8-oxo-dA is protonated at N1 with a p*K*_a_ of 2.9 (5). This p*K*_a_ would need to be elevated in the TDG active site to facilitate acid-catalyzed oxoA excision under physiological conditions.

Our results indicate an essential catalytic role for N140 in excision of oxoA, as observed previously for other TDG substrates. The N140A mutation reduces TDG activity by 11,300- to 29,500-fold for the four oxoA pairs, with the largest effect for T⋅oxoA. These mutational effects are similar in magnitude (>10^4^-fold) to those observed for other substrates. These findings suggest that N140 catalyzes oxoA excision through binding and positioning the nucleophilic water molecule, as observed in crystal structures of TDG bound to noncleavable analogs of several pyrimidine substrates (51, 52, 53). Our results also have implications regarding the feasibility of determining a crystal structure of N191A-TDG^82–308^ bound to DNA containing an oxoA base pair. Previous studies show that for one substrate, G⋅caC, the N140A mutation eliminates detectable glycosylase activity while preserving substrate binding. These conditions allowed us to solve a crystal structure of the stable enzyme–substrate complex comprising N191A-TDG^82–308^–bound G⋅caC DNA with ca-dC flipped into the active site. Observation that N140A-TDG^82–308^ retains significant oxoA activity, even for the least efficient substrate (T⋅oxoA) indicates that alternative approaches will be necessary for solving a structure of TDG with oxoA flipped stably into its active site.

### MUG lacks substantial oxoA activity

We find that *E. coli* MUG has no detectable activity for removing oxoA from T⋅oxoA pairs and that its activity is exceedingly low for G⋅oxoA, A⋅oxoA, and C⋅oxoA pairs. The vast difference in oxoA activity between bacterial MUG and mammalian TDG is quite remarkable, given that these orthologs have similar activity for G·U substrates and feature substantial (32%) amino acid sequence identity. However, TDG and MUG exhibit large differences in activity for other substrates. For example, activity for a G⋅T mispair is substantial for TDG but extremely low for MUG (48, 63). On the other hand, we find that activity for G·εC is 100-fold greater for MUG relative to TDG. Similarly, 1,*N*^2^-ethenoguanine is efficiently removed by MUG but not TDG (64). These large activity differences are in keeping with the fact that most active site residues differ between TDG and MUG. For example, residue N191 of TDG, which is critical for its excision of oxoA, is replaced by Lys in the corresponding position of MUG, as shown by structure-based alignment (59). Together, these findings suggest that TDG, unlike MUG, has evolved under selective pressure to efficiently excise oxoA from DNA.

## Experimental procedures

### Oligodeoxynucleotides and duplex DNA

Oligodeoxynucleotides (ODNs) were obtained from IDT or the Keck Foundation Biotechnology Resource Laboratory of Yale University. ODNs containing oxoA or εC (3,*N*^4^-ethenocytosine) were synthesized at Yale using phosphoramidites from Glen Research. The ODNs were purified by reverse-phase HPLC (46), and purity was assessed by anion-exchange HPLC under denaturing (pH 12) conditions using a DNAPac PA200 RS column (Thermo) (65). ODNs were exchanged into 0.01 M Tris–HCl (pH 8), 0.05 M NaCl, and 1 mM EDTA using centrifugal concentrators. ODN concentration was determined by absorbance (260 nm) using the pairwise extinction coefficient (45). DNA substrates (Fig. 2) were made by mixing a target and complementary strand, heating to 80 °C, and slowly cooling to room temperature.

### Enzyme purification

Full-length human TDG was expressed in bacteria and purified as described (45). The TDG^82–308^ construct and variants thereof (N140A, N191A) were also expressed in bacteria and purified as described (51). Enzyme purity was >99% as assessed by SDS-PAGE with Coomassie staining. *E. coli* MUG was produced in *E. coli* BL21(DE3) cells transformed with a pD441-SR expression plasmid (ATUM). Cells growing at 37 °C were induced with 1 mM IPTG, and protein expression was continued at 37 °C for 5 h. Purification of MUG was based on a previous report (66) using metal (Ni^2+^) affinity, ion exchange where protein was loaded through tandem Q-SP sepharose columns and eluted from the SP column, followed by size exclusion (S200) chromatography (all columns from GE Healthcare). Concentration of the purified enzymes was determined by absorbance at 280 nm (67, 68) using an extinction coefficient of ε^280^ = 25.6 mM^−1^ cm^−1^ for MUG, ε^280^ = 31.5 mM^−1^ cm^−1^ for TDG, and ε^280^ = 17.4 mM^−1^ cm^−1^ for TDG^111–308^ and TDG^82–308^ (and variants thereof).

### Glycosylase activity assays

The single-turnover reactions were initiated by adding enzyme to HEN.1 buffer (0.02 M Hepes [pH 7.5], 0.1 M NaCl, and 0.2 mM EDTA) containing a given DNA substrate (0.5 μM), at room temperature (23 °C). The enzyme concentrations used in the experiments (2.5–10 μM) were at least fivefold excess relative to substrate and are provided in the figure legends. Aliquots were removed and added to 10% (v:v) quench solution (1.0 M NaOH and 0.1 M EDTA) to immediately halt the reaction. As needed, a rapid quench-flow instrument (RQF-3; KinTek Corporation) was used to collect short reaction time points (<4 s). The quenched reaction samples were heated for 3 to 5 min at 85 °C to quantitatively cleave the DNA backbone at abasic sites, and the resulting DNA fragments were resolved by ultra HPLC. The integrals for peaks corresponding to intact and cleaved target strand were used to determine the fraction product for a given reaction sample (65). In all figures presenting a progress curve (fraction product *versus* time), the fraction product shown at a given time point represents the average, with error bars indicating standard deviation, obtained from at least three independent kinetics experiments. The rate constant (*k*_obs_) and amplitude (*A*) were obtained by fitting the progress curves to Equation 1 using nonlinear regression:

(1) $$\text{fraction}\;\text{product}=A\left(1-\exp\left(-k_{\text{obs}}t\right)\right)$$

where *A* is the amplitude, *k*_obs_ is the rate constant, and *t* is the reaction time. For reactions involving N191A-TDG^82–308^, the fitted amplitude is relatively low for some substrates, which could reflect reduced stability of this particular TDG variant. For some reactions involving N140A-TDG^82–308^, the progress curves were fitted to a linear equation, where *k*_obs_ is given by the slope, because the reactions were too slow to be fitted using Equation 1. The presence of saturating enzyme conditions ([E] >> *K*_*d*_) in the single-turnover reactions was evidenced by observation that the rate constants (*k*_obs_) were similar for multiple enzyme concentrations (typically, 2.5 and 5 μM). Previous studies show that TDG and TDG^82–308^ bind very tightly (*K*_*d*_ < 0.02 μM) to multiple DNA substrates, including G·T, G·U, G·fC, and G·caC (51, 67, 69). We used a 10 μM concentration of TDG^111–308^ and MUG to ensure saturating enzyme conditions (48, 51).

## Data availability

The data supporting the findings of this study are available within the article and the supporting information.

## Supporting information

This article contains supporting information.

## Conflict of interest

The authors declare that they have no conflicts of interest with the contents of this article.
