# Supplementary material for: Rapid excision of oxidized adenine by human thymine DNA glycosylase
Source: J Biol Chem. 2022 Nov 30;299(1):102756. doi: 10.1016/j.jbc.2022.102756 (PMC9800633; doi:10.1016/j.jbc.2022.102756)
Supplement: Supplemental Figures S1–S5 [file mmc1.pdf]

Supporting Information

Rapid Excision of Oxidized Adenine by Human Thymine DNA Glycosylase

Hardler W. Servius, Lakshmi S. Pidugu, Matthew E. Sherman, and Alexander C. Drohat

Department of Biochemistry and Molecular Biology, University of Maryland School of Medicine, Baltimore, MD 21201, United States

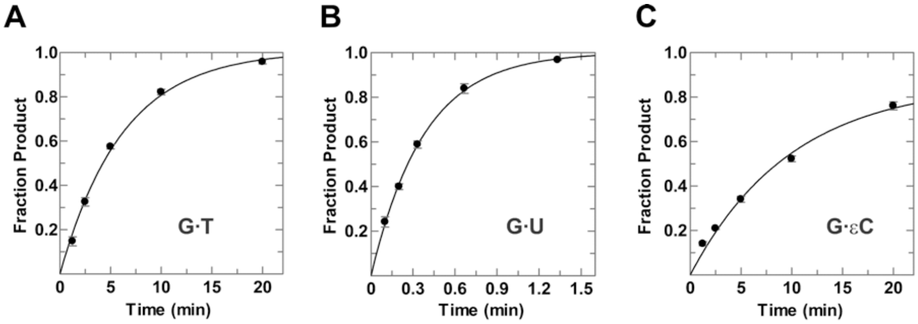

Figure S1. TDG activity for G·T, G·U, and G·εC substrates. TDG (2.5 μM) (A) excises T from G·T pairs with $k_{obs} = 0.16 \pm 0.01$ min^{-1}, (B) excises U from G·U pairs with $k_{obs} = 2.66 \pm 0.08$ min^{-1}, and (C) excises εC from G·εC with $k_{obs} = 0.10 \pm 0.01$ min^{-1}.

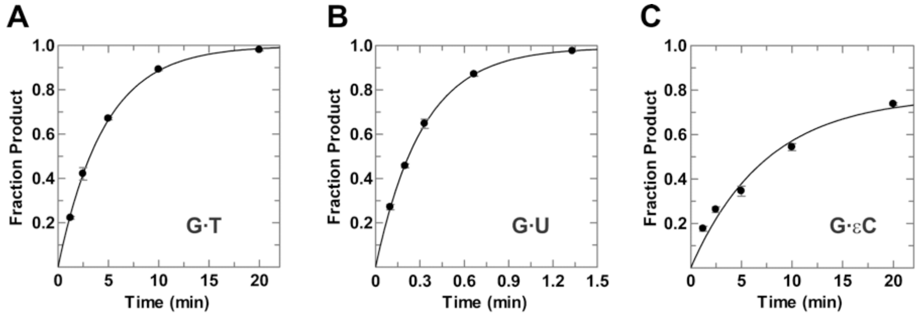

Figure S2. TDG$^{82\text{-}308}$ activity for G·T, G·U, and G·εC substrates. TDG$^{82\text{-}308}$ (2.5 μM) (A) excises T from G·T pairs with $k_{obs} = 0.22 \pm 0.01$ min^{-1}, (B) excises U from G·U pairs with $k_{obs} = 3.15 \pm 0.06$ min^{-1}, and (C) excises εC from G·εC pairs with $k_{obs} = 0.14 \pm 0.02$ min^{-1}.

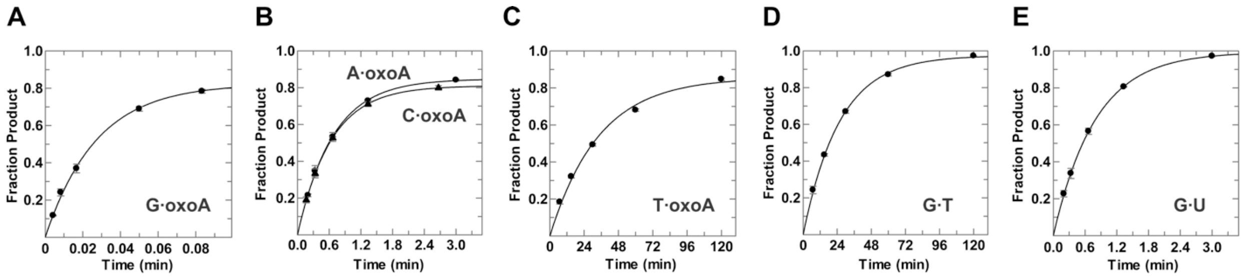

Figure S3. TDG$^{111-308}$ activity for x·oxoA pairs and for G·T and G·U substrates. TDG$^{111-308}$ (10 μM) (A) processes G·oxoA pairs with $k_{obs} = 36.7 \pm 1.2$ min^{-1}, (B) A·oxoA pairs with $k_{obs} = 2.08 \pm 0.07$ min^{-1} (circles) and C·oxoA pairs with $k_{obs} = 1.58 \pm 0.05$ min^{-1} (triangles), (C) processes T·oxoA pairs with $k_{obs} = 0.029 \pm 0.001$ min^{-1}, (D) processes G·T pairs with $k_{obs} = 0.039 \pm 0.001$ min^{-1}, and (E) acts on G·U pairs with $k_{obs} = 1.26 \pm 0.03$ min^{-1}.

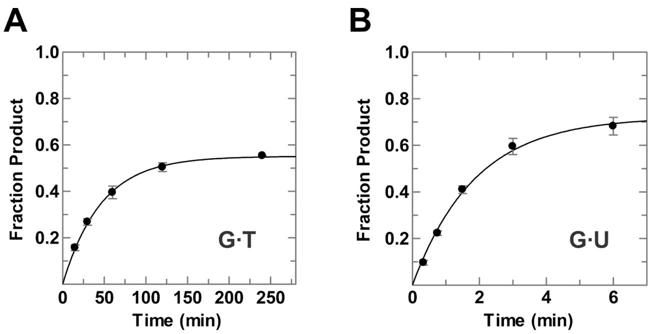

Figure S4. N191A-TDG$^{82-308}$ activity for G·T and G·U substrates. N191A-TDG$^{82-308}$ (5 μM) (A) excises T from G·T pairs with $k_{obs} = 0.022 \pm 0.001$ min^{-1}, and (B) excises U from G·U pairs with $k_{obs} = 0.53 \pm 0.04$ min^{-1}.

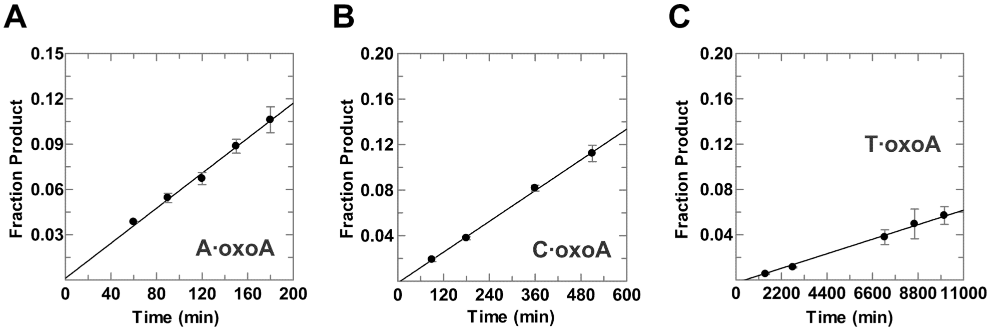

Figure S5. N140A-TDG$^{82-308}$ activity for excising oxoA from DNA. N140A-TDG$^{82-308}$ (2.5 μM) (A) processes A·oxoA pairs with $k_{obs} = (5.8 \pm 0.2) \times 10^{-4}$ min^{-1}, (B) acts on C·oxoA pairs with $k_{obs} = (2.3 \pm 0.1) \times 10^{-4}$ min^{-1}, and (C) processes T·oxoA pairs with $k_{obs} = (5.9 \pm 0.4) \times 10^{-6}$ min^{-1}. For these reactions involving N140A-TDG$^{82-308}$, the progress curves were fitted to a linear equation, where k_{obs} is given by the slope, because the reactions were too slow to be fitted using eq. 1.
